# Metaversum: Hype oder Hoffnung?

**DOI:** 10.1365/s35764-023-00459-1

**Published:** 2023-03-29

**Authors:** Martha Boeckenfeld, Hartmut Feucht, Markus Schröder

**Affiliations:** Hochschule Fresenius Online Plus GmbH, Köln, Deutschland

„Am Metaversum wird künftig keiner mehr vorbeikommen.“ Ist dem wirklich so? Jetzt, wo wir bereits viele Anwendungsfälle sehen und den Aufstieg des Metaversums erleben, wird das Metaversum realer werden als je zuvor.

Noch hat das Metaversum seinen endgültigen Zustand nicht erreicht – es ist aber nicht nur eine Technologie, sondern zeigt vielmehr auf, wie wir unser Leben und unsere Zukunft gestalten können – eine Fusion der digitalen und realen Welt. Stellen wir uns vor, es ist das Jahr 2030 und wir beobachten die Marslandung mit XR (Extended Reality) und stehen bei der Landung direkt neben dem Astronauten. Wir leben in virtuellen Welten, zahlen mit einer digitalen Währung, digitales Eigentum ist Teil des Testaments. Was ist unsere Rolle und die von Unternehmen in der neuen Welt? Wir nähern uns der Zukunft, indem wir zunächst eine Auslegeordnung definieren, wo stehen wir heute, was sind die Anwendungsfälle? Mit einem Fokus auf das Bildungswesen (schulische und universitäre Aus- und Weiterbildung), das kollaborative Arbeiten und das Gesundheitssystem, betrachten wir Anwendungsfälle, die bereits in den nächsten 3–5 Jahren von hoher Bedeutung sein werden, und schauen diese im Kontext der Risiken und Chancen an.

## Was ist das Metaversum?

Das Wort Metaversum kommt von den Worten meta (in der Bedeutung „jenseits“) und Universum. Geprägt wurde der Begriff 1992 von Neal Stephenson in seinem Science-Fiction-Roman Snow Crash, der eher ein „dystopian“ Metaversum zeichnet. Eine eindeutige Definition des Metaversums existiert derzeit nicht. Vielmehr gibt es Diskussionen, was das Metaversum sein könnte, was es nicht ist, was es sein sollte bzw. sein muss und was zu ihm gehört. Fragt man Matthew Ball, dessen Essays zum Metaverse 2020 hohe Wellen schlugen, so ist das Metaversum die Zukunft des Internets, ein „expansives Netzwerk aus persistenten, in Echtzeit gerenderten 3‑D-Welten und Simulationen, das Kontinuität von Identitäten, Objekten, Geschichtlichkeit, Währungen und Berechtigungen bietet und sich synchron von unbegrenzt vielen Nutzern auf individuelle Art und Weise erfahren lässt“ [1].

**Figure 1:**
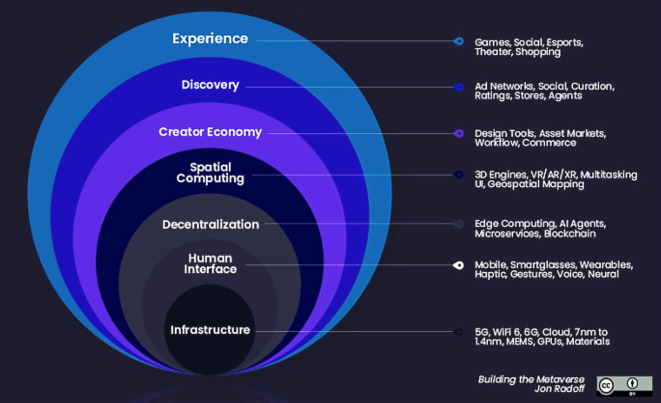

Was heißt das aber konkret und woraus „besteht“ dann eigentlich das Metaversum? Eine gute Übersicht, auf welchen Ebenen das Metaversum beruht, liefert der sehr lesenswerte Artikel „The Metaverse Value-Chain“ von Jon Radoff [2]. Anschaulich stellt der Autor in einer Grafik die „Seven Layers of the Metaverse“ und damit die Wertschöpfungskette des Metaversums vor (Abb. 1).

Für viele ist das Metaversum jedoch immer noch unsichtbar. Ist es nur ein weiterer Marketing-Gag für bestimmte Großunternehmen? Oder wird es das nächste große Ding in der Geschichte der Menschheit sein?

Und trotz der Nebulosität dieser Welt sehen manche Experten schon klare Chancen für einen Erfolg: Das US-amerikanische Marktforschungsunternehmen Gartner geht in seiner Prognose davon aus, dass bis zum Jahr 2026 schon 25 % der Menschen mindestens eine Stunde pro Tag im Metaversum verbringen [3]. Laut KPMG werden 70 % der Marken bis 2027 im Metaversum vertreten sein [4]. Die Bank HSBC erwartet, dass bis 2027 insgesamt 700 Mio. Menschen Augmented Reality/Virtual Reality (AR/VR) nutzen [5]. McKinsey schätzt, dass das Metaversum bis zum Jahr 2030 bis zu 5 Billionen US-Dollar einbringen könnte – eine beträchtliche Chance, die durch die rasche Akzeptanz immersiver virtueller Umgebungen durch die Verbraucher, die zunehmende Offenheit für digitale Güter und neue Technologien sowie durch umfangreiche Investitionen des Privatsektors entstehen wird [6]. Einer umfassenden Akzeptanz stehen immer noch viele Herausforderungen entgegen und nicht alle Ideen werden erfolgreich oder in ihrer aktuellen Form überhaupt umsetzbar sein. Technische Fortschritte sind nötig, um High-Fidelity-Grafiken, niedrige Latenz und ein robustes Volumen zu erschwinglichen Preisen bereitstellen zu können. Wie beim jetzigen Internet, werden dieselben regulatorischen Bedenken wie etwa Datenschutz und Datensicherheit, mit denen die Plattformen der großen Technologieunternehmen heute schon konfrontiert sind, auch das Metaverse betreffen.

## Der digitale Shift

**Figure 2:**
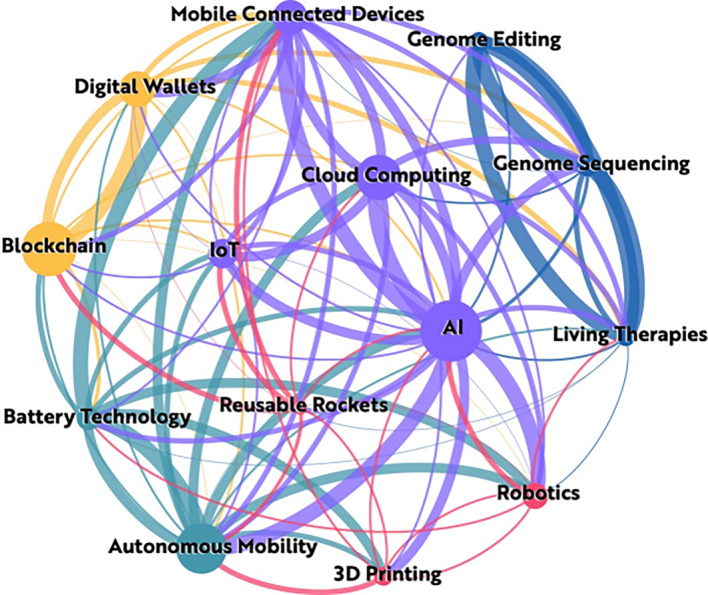

Die letzten 5 Jahre waren geprägt von einer digitalen Disruption, die wir vorher noch nicht so erlebt haben. Die COVID-19-Pandemie hat einen Shift zu mehr Digitalisierung ausgelöst, die alle unsere Lebensbereiche betrifft und unsere Gewohnheiten und Präferenzen verändert hat. Wir verbringen bereits mehr und mehr Zeit online, bis 2030 erwarten Experten, dass wir 52 % unserer freien Zeit online und nur noch 48 % offline sein werden [7]. Zeitgleich konvergieren Technologien und wachsen mit exponentieller Kraft, von wiederverwertbaren Raketen bis hin zur Genbearbeitung, Proteinfaltung, autonomen Fahrzeugen oder digitalen und virtuellen Agenten. Diese Disruption erfolgt durch die rasante Entwicklung der Technologien Artificial Intelligence (AI), Edge Computing, Cloud, Blockchain, Virtual Reality, 5G, Robotics und IoT (Internet of Things), die miteinander zur gleichen Zeit verschmelzen (Abb. 2). Das Zahlen mit einer digitalen Wallet ist seit 2017 eine weitere Alternative als Zahlungsmittel und seit 2021 sind mehr Zahlungen im E‑Commerce-Bereich am Point of Sale mittels einer digitalen Wallet erfolgt als mit einer Visakarte (Visa Europe Management Services Limited, London, UK) – sicherlich ein Effekt, der der Covid-19 Pandemie geschuldet wurde.

„Wir glauben, dass Historiker auf diese Zeit zurückblicken werden als eine Ära von beispielloser technologischer Entwicklung und sie sagen werden: alles hat sich verändert“ [7].

Daher sprechen wir bereits heute von einer vierten Computerrevolution – das Metaversum wird das Internet nicht ersetzen, sondern eher eine Weiterentwicklung sein, wie auch das mobile Internet sich von 1960 bis 1990 weiterentwickelt hat.

## Heutige Anwendungsfälle im Metaversum

Wir sehen im Bildungswesen (schulische und universitäre Aus- und Weiterbildung), das kollaborative Arbeiten und das Gesundheitssystem als Anwendungsfälle, die bereits in den nächsten 3–5 Jahren von hoher Bedeutung sein werden.

Aber wo werden wir in den nächsten Jahren konkreten Nutzen durch das Metaverse sehen? In einer Umfrage unter US-amerikanischen Entwicklern wurde deutlich, dass diese den größten Nutzen des Metaverse im Bereich des Gamings und des Entertainments sehen werden [8]. Aber die dort entwickelten Technologien können auch in anderen Bereichen wertstiftend eingesetzt werden. Dazu möchten wir hier auf die Bereiche der Zusammenarbeit, der Medizin und der Aus- und Weiterbildung eingehen.

Durch die Coronapandemie hat die Zusammenarbeit im virtuellen Raum einen großen Aufschwung erlebt. Hier dominiert zwar noch die zweidimensionale Kommunikation über Videokonferenztools. Aber es gibt Ansätze, ganze Veranstaltungen mit Vorträgen, Breakout-Sessions und auch Small-Talk-Möglichkeiten in die virtuelle Welt zu überführen. Noch sind diese in der breiten Nutzung eher im zweidimensionalen Netz zu finden, was aber auch an der noch geringen Verbreitung der entsprechenden Devices liegt. Der nächste Schritt wird aber eindeutig das Eintauchen in das Metaverse für diese Art der Zusammenarbeit sein. Es ist ein entscheidender Unterschied, ob man zu Hause vor einer Webcam sitzt und mit seinen Augen das gewohnte Umfeld des Homeoffice wahrnimmt oder ob man mithilfe einer 3‑D-Brille vollständig in den virtuellen Workshop abtaucht.

**Figure 3:**
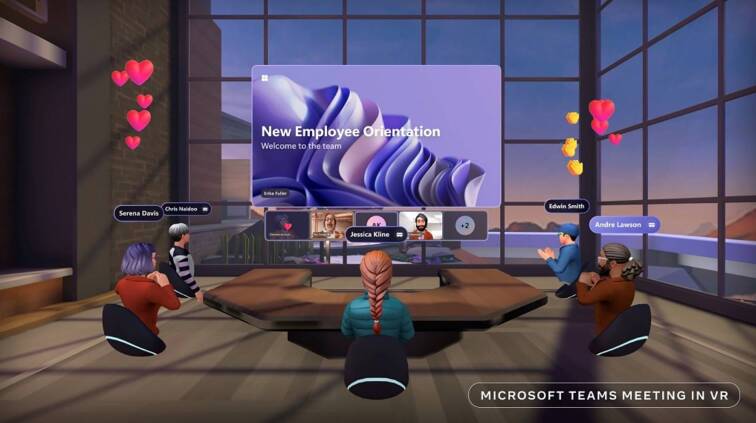

Die Kooperation zwischen Microsoft und Meta sorgte kürzlich für Schlagzeilen [9]. So sind Anwendungen wie Microsoft Teams (Microsoft Corporation, Redmond, Washington, USA) }in die Horizon Workrooms von Meta (Meta Platforms Inc., Menlo Park, California, USA) integriert, und auch die gängigen Microsoft-365-Produkte (Microsoft Corporation, Redmond, Washington, USA), wie Word, Outlook und Excel, lassen sich im Metaverse nutzen. Es wird erwartet, dass diese Partnerschaft zu einer Steigerung der Nutzerzahlen im Metaverse führt, schließlich erlaubt sie 70 Mio. monatlich aktiven Nutzern von Microsoft Teams, im virtuellen Raum Meetings zu führen und mit Teammitgliedern in Mixed Reality und Virtual Reality zusammenzuarbeiten (Abb. 3).

Ebenso gibt es bereits viele Anwendungsfälle in der Medizin. Mithilfe des Metaversums können angehende Chirurgen unter fast realistischen Bedingungen trainieren. Dies ist ein Ansatz, den wir aus anderen Bereichen schon länger kennen. Piloten trainieren in Flugsimulatoren. In diesen Simulatoren erhalten sie den Eindruck einer realen Welt. Diese Simulatoren bilden das komplette Cockpit eines Flugzeugs ab und die Sicht nach außen wird durch Grafiken simuliert. Im Gegensatz zu VR-Brillen und -Handschuhen wird hier eine detailgetreue Abbildung eines Cockpits genutzt. Bei der Ausbildung von Chirurgen kommt hinzu, dass die Darstellung in VR realistischer wirkt als die Arbeit mit anderen Mitteln.

**Figure 4:**
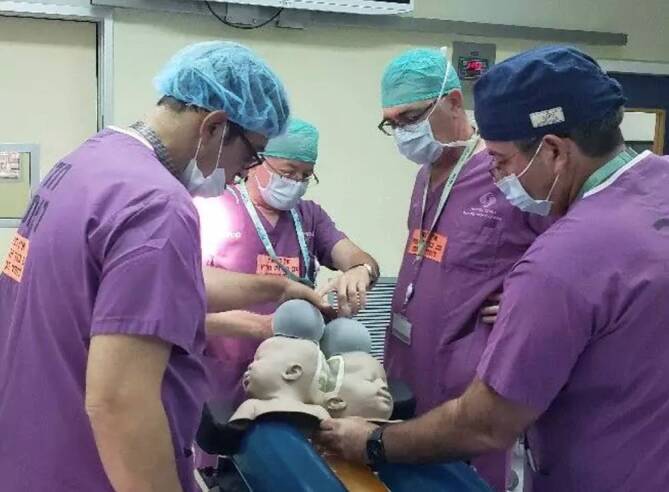

Eine weitere Möglichkeit ist die Unterstützung von (noch) unerfahrenen Operateuren durch Experten, die die Operation per VR verfolgen [10]. Der Vorteil liegt auf der Hand, der Experte kann viel mehr Operationen begleiten, als wenn er zu jeder physisch reisen müsste. Aber hier werden auch direkt Herausforderungen bzw. Anforderungen an die Infrastruktur deutlich. Während in einer klassischen Videokonferenz kurze Aussetzer noch akzeptabel sein mögen, ist dies bei einer Operation nicht so. Schließlich geht es um das Leben eines Patienten. Hier wird auf den modernen 5‑G-Standard gesetzt, welcher Verzögerungen in der Übertragung drastisch reduziert. Im August dieses Jahrs ist es einem Team an Ärzten aus Israel nach monatelanger Vorbereitung auch mithilfe von Virtual Reality und zahlreichen 3‑D-gedruckten Modellen gelungen, eine äußerst selten vorkommende Variante siamesischer Zwillinge zu trennen. Die 3‑D-Technologien verhalfen den Ärzten zu umfangreichen Möglichkeiten, wirklich jeden einzelnen Punkt der Operation trainieren zu können. Die mit 3‑D-Druck hergestellten Modelle und die Möglichkeit, sich mit Experten aus London auszutauschen, waren dabei ein wichtiger Konvergenzpunkt für die Mediziner ([11]; Abb. 4).

**Figure 5:**
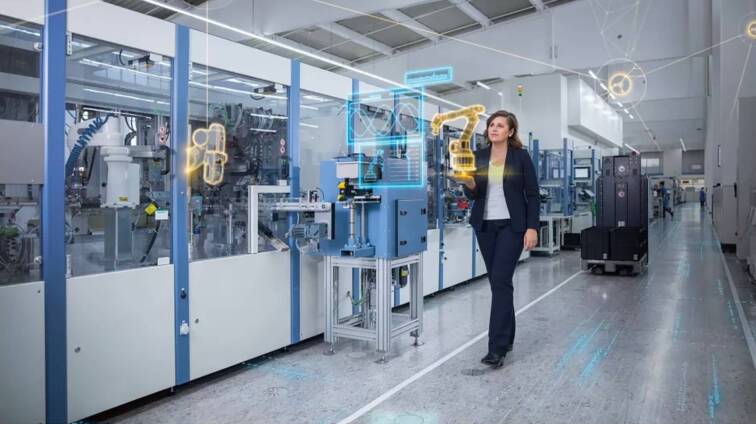

Im Bereich der Aus- und Weiterbildung sehen wir weitere deutliche Anwendungsfälle für das Metaverse. Wir haben gerade den Flugsimulator diskutiert, welcher leider aufgrund der aufwendigen Hardware sehr kostspielig ist. Durch die Nutzung von VR-Hardware wird die Simulation von anderen Arbeitsplätzen für die Ausbildung von Fachkräften effizient und kostengünstiger möglich. In der Industrie sind derzeit noch die schriftlichen Arbeitsanweisungen der Standard. Mittels Metaverse würde es möglich, diese erlebbar zu machen. Kein langes Lesen wäre mehr nötig, sondern im firmeneigene Metaverse erlebt man den Arbeitsplatz mit den zugehörigen Prozessen. Im „industriellen Metaversum“ werden bereits digitale Zwillinge mit den virtuellen Welten zur Verbesserung und Vereinfachung von Prozessen eingesetzt. Die Verbindung von Siemens Xcelerator (Siemens AG, München, Deutschland) und NVIDIA Omniverse (Nvidia Corporation, Santa Clara, California, USA) läutet ein neues Zeitalter der industriellen Automatisierung ein. Damit können Industrieunternehmen jeder Größe Closed-Loop- und digitale Zwilling mit vollständiger Detailtreue in Echtzeit erstellen ([12]; Abb. 5).

## Chancen und Herausforderungen

Es ist schon etwas schwierig Chancen und Herausforderungen von etwas zu diskutieren, von dem wir noch nicht wissen, wohin es sich entwickeln wird. Es ist in etwa so, als hätte man vor 30 oder gar 50 Jahren die Chancen und Risiken des Internets zu fassen versucht. In der rasanten Entwicklung der Digitalisierung sind viele Anwendungsmöglichkeiten der digitalen Welt nicht im Vorhinein denkbar gewesen.

Es gibt aber dennoch schon einige Bereiche, für die wir bereits heute Aussagen zu Chancen bzw. Herausforderungen treffen können. Zunächst wird uns allen einleuchten, dass das Metaverse neue Anforderungen an die Internetbandbreite und die Reaktionszeiten stellen wird. Durch die aufwendige, realitätsnahe Darstellung von Räumen, Landschaften aber auch der Avatare werden hohe Rechenleistungen notwendig sein. Um die Interaktion auch wirklich real zu gestalten, muss die Latenzzeit zwischen meiner Aktion und deren Übertragung ins Metaverse und damit zu den anderen Usern für den Menschen nicht feststellbar sein.

Eine weitere Frage, die heute noch nicht beantwortet werden kann, ist, ob es ein Metaversum geben wird [8, 13], in dem sich Galaxien einzelner Unternehmen wie Meta, Microsoft oder Roblox oder anderer Akteure befinden, zwischen denen man aber einfach hin und her wechseln kann. Dies hätte den Vorteil für den Nutzer, dass er nicht immer wieder an Grenzen stößt und sich dann mit neuen Daten in ein anderes „Metaverse“ einloggen muss. Wenn das Metaverse eine Verknüpfung zur realen Welt haben soll, dann sollte unsere Forderung sein, dass man zwischen den Galaxien der Metaverse-Akteure einfach wechseln kann und seine digitalen Erfahrungen mitnehmen kann. Auch in der realen Welt muss man seinen Erfahrungsschatz nicht an der Grenze zum Nachbarland zurücklassen. Das Gleiche gilt für die Bezahlmöglichkeit. Wird es eine oder mehrere universelle Metawährungen geben?

Eine weitere prominente Thematik wird sich um den Umgang mit Daten und mit der Sicherheit an sich im Metaverse befassen. Hier spielen nicht nur die persönlichen Vorstellungen der einzelnen Nutzer, sondern auch die Anforderungen legislativer Natur eine Rolle. Gerade die letzteren sind in den verschiedenen Regionen der Welt doch stark unterschiedlich. Es wird einen großen Aufwand bedeuten, diese Anforderungen in allen Ländern abzudecken und zu aktualisieren. Wird die Kommunikation im Metaverse genauso vergänglich sein, wie der Plausch mit dem Nachbarn? Oder wird jede Interaktion gespeichert, ausgewertet und monetarisiert?

Dies führt direkt zur nächsten Thematik. Wie wird das Metaverse „regiert“ bzw. welche Regeln gelten im Metaverse? Hier wird auch der Begriff der Metaverse Governance genutzt. Diese Governance ist zu definieren und muss letztlich die Zustimmung der Nutzer erfahren, sonst wird die Akzeptanz der Nutzer fehlen.

Einige der genannten Themenkomplexe laufen Gefahr, die Inklusion und Gerechtigkeit im Metaverse zu gefährden. Denken wir doch einmal an die „Devices“, die für die Teilnahme der User am Metaverse notwendig sind. Wer stellt sicher, dass es diese auch für benachteiligte Gruppen der Gesellschaft geben wird? Die Benachteiligung kann hier nicht nur wirtschaftlicher Natur, sondern auch körperlicher Natur sein. Ein weiteres Beispiel ist die benötigte Bandbreite für die Datenübertragung. Wenn wir schon in entwickelten Ländern Schwierigkeiten haben ausreichend Bandbreite für etablierte Internetanwendungen zur Verfügung zu stellen, wie soll dies bei den deutlich höheren Anforderungen des Metaverse funktionieren? Von ländlichen Regionen in der dritten Welt sprechen wir besser gar nicht.

Es sind aber auch noch andere Punkte zu berücksichtigen. Meta [14] wirbt für die Möglichkeit, Lernende in die Geschichte eintauchen zu lassen. Es ist sicherlich eine spannende Erfahrung, Mark Anton in Rom in einer Debatte zu erleben. Aber diese Art der Darstellung der Geschichte birgt auch Gefahren. Wir wissen heute bereits, welche Macht das geschriebene Wort hat. Ein Adjektiv an der richtigen Stelle sorgt für einen entsprechenden Impuls beim Lesenden. Jetzt haben wir im Metaverse aber alle Möglichkeiten der AR/VR. Dies bedeutet, dass Personen nicht über gesprochene Worte – die eventuell aus einem Protokoll stammen –, sondern als agierende Personen dargestellt werden. Das heißt Mimik, Aussehen, Stimme, Kleidung – alle Komponenten der verbalen und nonverbalen Kommunikation kommen zum Einsatz. Einzig der Geruchssinn bleibt unangetastet. Die Versuchung wird groß sein, einen vermeintlichen Bösewicht der Geschichte auch als einen solchen in bester Hollywoodmanier darzustellen. Aber ist es auch wirklich so gewesen? Wird Geschichte hier authentisch, objektiv erfahren oder schlägt die Propagandamaschinerie nach George Orwell hier voll zu? Die Anforderung an den Lernenden, das Gesehene oder besser Erfahrene oder Erlebte kritisch zu hinterfragen, bleibt bestehen. Im Übrigen erleben wir bereits in den jetzigen sozialen Medien intensive Propaganda und Desinformation.

Als Letztes bleibt noch die Frage wie sich das Metaverse bzw. dessen intensive Nutzung auf unsere Gesundheit auswirken wird. Bislang gibt es keine Studien, die einen negativen gesundheitlichen Einfluss von Mobiltelefonen belegen [13]. Für die bislang noch nicht verfügbaren Devices, die für das ganzheitliche Eintauchen ins Metaverse notwendig sind, kann dementsprechend noch keine Aussage getroffen werden. Auch bleibt die Frage bestehen, wie unser Körper auf den laufenden Wechsel zwischen realer Welt und Metaverse reagieren wird.

### Zusammenfassung

Das Metaverse wird kommen.

Es gibt bereits Anwendungen.

Das Metaverse bietet Chancen und Herausforderungen.

Viele Möglichkeiten übersehen wir heute noch nicht

Das Metaverse ist mehr als ein Hype

Das Metaverse schafft immersive Erlebnisse

### Handlungsempfehlung

Besser jetzt ins Metaverse einsteigen, als die Entwicklung zu verschlafen.

Kritisch mit den Chancen und Herausforderungen umgehen.

Offen sein für Möglichkeiten, die wir heute noch nicht abschätzen können.
